# Mesenchymal stem cell-derived exosomes: a new hope for improving asthma airway remodeling

**DOI:** 10.1080/07853890.2025.2591447

**Published:** 2025-11-27

**Authors:** Rui Suo, Tianxu Hao, Ruxianguli Wumaier, Jingjing Zhao, Xintao Du, Ying Suo, Xiaoyun Zhao

**Affiliations:** ^a^ Chest Hospital, Tianjin University, Tianjin, China; ^b^ Clinical School of Thoracic, Tianjin Medical University, Tianjin, China; ^c^ Department of Respiratory & Critical Care Medicine, Tianjin Chest Hospital, Tianjin, China

**Keywords:** Mesenchymal stem cells, exosomes, asthma, airway remodeling

## Abstract

**Background:**

Asthma features airway hyperresponsiveness, reversible airflow obstruction, and airway remodeling. Its rising incidence and mortality confer a growing societal burden. Recent studies have revealed the therapeutic potential of mesenchymal stem cell-derived exosomes (MSC-EXOs) for asthma, warranting further investigation into their underlying mechanisms and clinical implications.

**Objective:**

This review aims to provide a comprehensive summary of the application and underlying mechanisms of MSC-EXOs in mitigating airway remodeling in asthma, with the goal of exploring the feasibility of MSC-EXOs as a potential therapeutic strategy for asthma management.

**Methods:**

A comprehensive literature review was conducted using the keywords “Asthma,” “Airway Remodeling,” “Mesenchymal Stem Cells,” and “Exosomes.” Relevant studies published between March 17, 2000, and September 24, 2024, were identified through systematic searches in PubMed, Embase, and Web of Science. The retrieved studies were systematically analyzed and organized to offer a thorough and comprehensive overview of the relevant literature.

**Results:**

This review offers a detailed examination of the pathogenesis of asthma, current therapeutic strategies, and their inherent limitations. It delves into the sources, biological functions, and therapeutic potential of MSCs and MSC-EXOs in mitigating airway remodeling in asthma, along with the molecular mechanisms implicated in these processes. Furthermore, it identifies the limitations of current research on MSC-EXOs therapy for asthma and suggests promising directions for future investigations.

**Conclusion:**

This study emphasizes the advantages of MSC-EXOs as a therapeutic modality in the context of regenerative medicine for asthma treatment. Preclinical studies have consistently demonstrated the therapeutic potential of MSC-EXOs, suggesting that they may provide additional treatment options for asthma patients in the future.

## 1. Introduction

Asthma is a heterogeneous disease influenced by epigenetic regulation and environmental factors, characterized by chronic airway inflammation and airway hyperresponsiveness (AHR). Airway remodeling involves processes like thickening of the basement membrane, deposition of extracellular matrix (ECM), and proliferation of airway smooth muscle cells (ASMCs), contributing to asthma progression. Clinical features of asthma primarily manifest as recurrent wheezing, breathlessness, chest tightness, or cough [1], often accompanied by variable expiratory airflow limitation. Asthma is an increasingly common disease worldwide, affecting 300 million people worldwide, and has worsened in recent years due to demographic and environmental issues [2]. Current asthma treatments, including anti-inflammatory agents, glucocorticoids, and long-acting β2-adrenergic receptor agonists, can slow disease progression but do not reverse pulmonary damage or improve patients’ quality of life [3–6]. Hence, there’s a need for a further understanding of asthma’s molecular mechanisms and the development of novel therapeutic approaches.

Growing evidence indicates that Mesenchymal stem cells (MSCs) hold significant promise in pulmonary regenerative medicine and cell therapy due to their multidirectional differentiation potential, self-renewal capabilities, the ability for long time *in vitro* proliferation and paracrine effects, and immunoregulatory properties [7–10]. Notably, research highlights that the favorable functions of MSCs are primarily linked to their paracrine factors, especially extracellular vesicles (EVs) [11,12]. These EVs, mainly encompassing exosomes (EXOs), and some other particles, contain diverse miRNAs and have emerged as potential treatments for various pulmonary diseases. They act as signaling molecules facilitating communication between cells, particularly between epithelial cells and the pulmonary microenvironment [13–15]. In comparison to parent MSCs, EVs offer enhanced safety and the potential for storage without loss of function [16], opening new avenues for diagnosing and treating asthma. Recent studies have underscored the potential of extracellular vesicle (EV)-based therapies as a promising approach for asthma management. Bandeira et al. demonstrated that EV-mimetic nanovesicles (NVs), engineered to replicate human mesenchymal stem cell (MSC)-derived EVs, significantly reduced eosinophilic infiltration and cytokine production in bronchoalveolar lavage fluid (BALF) and lung tissue, thereby mitigating airway inflammation in ovalbumin (OVA)-induced asthma models [17]. Similarly, Xu et al. reported that the aerosolized administration of hypoxia-preconditioned human umbilical cord-derived mesenchymal stem cell exosomes (hUCMSC-EVs) effectively suppressed pulmonary inflammation and prevented airway remodeling in ovalbumin (OVA)-induced asthma models [18]. Furthermore, a meta-analysis indicated that MSC-derived microvesicles (MSC-MVs) modulate the immune system, attenuate inflammatory responses, and inhibit airway remodeling, underscoring their potential as a viable alternative therapeutic strategy for asthma management [19].

This review offers an exhaustive overview of the pathogenesis, treatment strategies, and recent advancements in asthma research, with a particular emphasis on the characteristics, sources, and biological functions of MSC-EXOs. The review further delves into the specific molecular mechanisms through which MSC-EXOs modulate airway remodeling in asthma and critically examines their potential as a therapeutic approach. Finally, the current limitations of existing studies are systematically summarized, and potential directions for future research are proposed.

## 2. Methods

### 2.1. Literature search strategy

To undertake a comprehensive and systematic literature review, we queried three major databases: PubMed, Embase, and Web of Science. Our search was focused on publications from March 17, 2000, to September 24, 2024, ensuring the inclusion of the most up-to-date research pertinent to this topic. The review employed an array of keywords pertaining to the potential of MSC-EXOs in ameliorating airway remodeling in asthma. These keywords included “Asthma”, “Airway Remodeling”, “Inflammation”, “Airway Hyperresponsiveness”, “Tissue Repair”, “Mesenchymal Stem Cells” and “Exosomes.” The use of these keywords in diverse combinations ensured a comprehensive search of the relevant literature.

### 2.2. Study selection

The initial search resulted in a collection of articles, which were first screened by reviewing their titles and abstracts to assess their relevance to the topic of MSC-EXOs therapy for asthma. The selected articles subsequently underwent a full-text review to confirm their relevance and evaluate their quality. The included materials consisted of original research articles, review papers, and conference proceedings. No geographical restrictions were imposed; however, all included articles were required to be published in English to ensure clarity and applicability in a broader context.

### 2.3. Inclusion and exclusion criteria

The inclusion criteria were as follows: (a) articles related to the use of MSC-EXOs in the treatment of asthma published in peer-reviewed journals, including clinical, cellular, and animal studies; (b) studies published in English.

The exclusion criteria were: (a) non-original articles, such as abstracts and letters to the editor; (b) studies not written in English; (c) duplicate studies; (d) reviews or veterinary research focused on dogs, cats, or horses; (e) articles with insufficient data.

## 3. Asthma

### 3.1. The mechanisms of asthma

**Figure 1.**
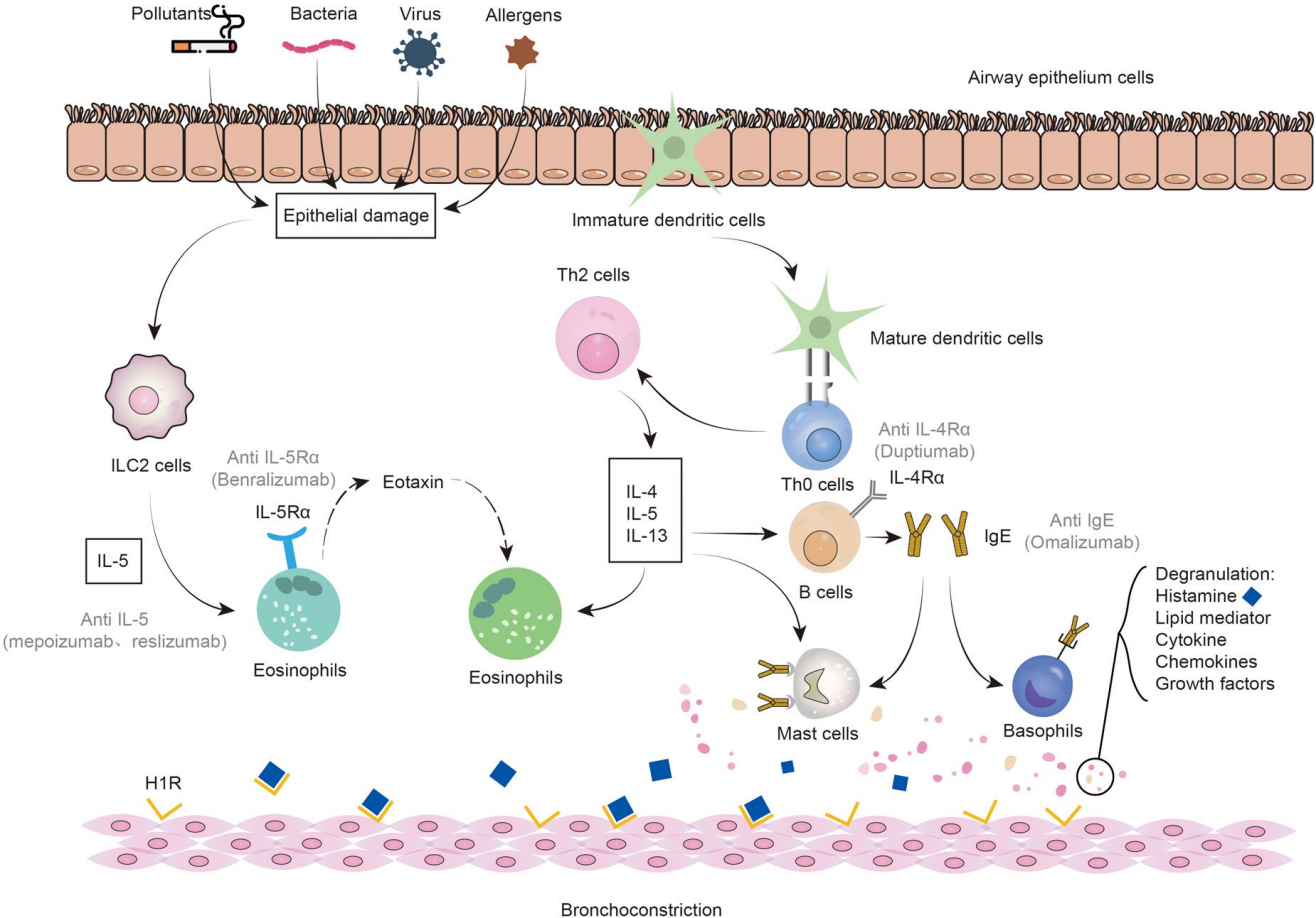
Cellular mechanisms of airway inflammation and airway remodeling in asthma.

Asthma, marked by airway inflammation, AHR, and airway remodeling, has become a major global public health issue. It represents a diverse inflammatory airway disease with intricate pathophysiological mechanisms [20] (Figure 1). While our comprehension of asthma phenotypes has grown in recent years, the precise mechanisms and pathways driving asthma’s development remain elusive. This review primarily delves into the latest progress in the mechanisms and treatment of allergic asthma. Allergic inflammation begins with dendritic cells (DCs) becoming activated in response to allergens. These activated DCs undergo maturation *via* major histocompatibility complex (MHC) class II molecules, presenting processed allergen polypeptide to naïve T(Th0) cells. DCs release chemotactic factors and induces Th0 to T-helper 2 (TH2) cells differentiation [21]. TH2 cells, in turn, secrete IL-13 and IL-4, which stimulate B lymphocytes to produce IgE. The IgE binds with high affinity to receptors on mast cells (MCs) and basophils, initiating a process called as “sensitization.” Upon subsequent exposure to allergens, MCs release three kinds of bioactive substances: cytoplasmic granules (such as histamine, a process known as “degranulation”), cytokines, and lipid mediators (comprising prostaglandins and leukotrienes). These events lead to vascular dilation and permeability changes, airway smooth muscle contraction, airflow obstruction, and mucus secretion, ultimately contributing to asthma symptoms [22]. Lipid mediators promote inflammation, encouraging immune cell infiltration, and inducing excessive mucus production. Notably, leukotrienes (LTS) also can promote mucus secretion and airway inflammation [23]. Additionally, sphingolipids and prostaglandins play roles in airway inflammation in asthma patients [24–27]. TH2 cells produce IL-5, a key factor in eosinophil differentiation and activation [28]. The increase of eosinophils is linked to frequent exacerbations and persistent airflow obstruction in asthma [29]. Asthma is characterized by the coordinated increase in IgE production induced by IL-4 and the rise in eosinophils induced by IL-5, both releasing granular substances that drive inflammation and bronchoconstriction [30]. With repeated exposure to allergens, both innate immune cells (such as eosinophils, MCs, basophils, neutrophils, etc.) and adaptive immune cells (including T lymphocytes and B lymphocytes) move to the site of inflammation. Subsequently, complex interactions ensue among innate immune cells, adaptive immune cells, and pulmonary structural cells, leading to bronchoconstriction, severe airway narrowing, and airway remodeling.

**Figure 2.**
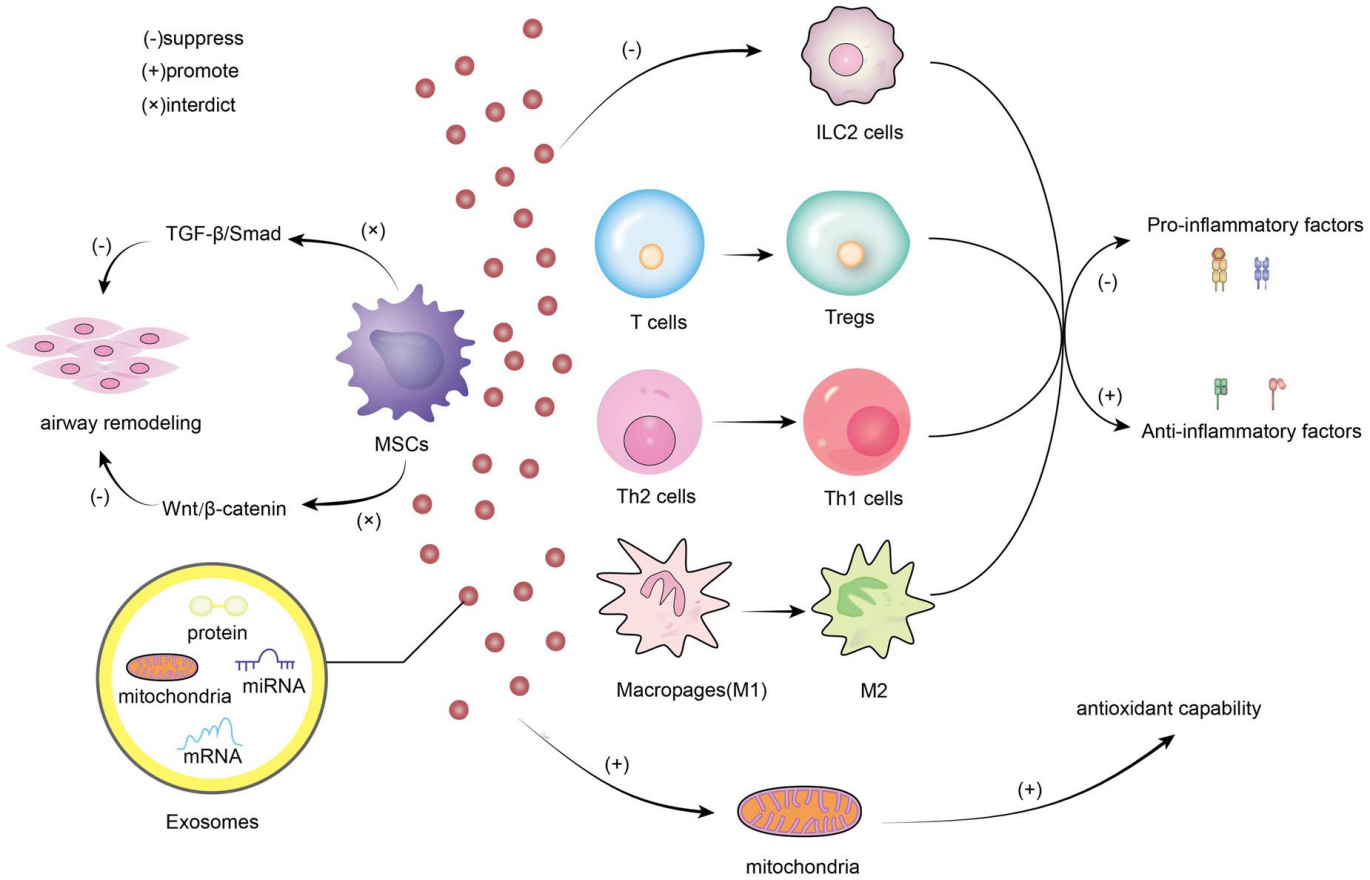
Mechanisms of Mesenchymal Stem Cell-Derived Exosomes in Ameliorating Asthmatic Inflammation and Airway Remodeling.

### 3.2. Asthma treatment research advances

Currently, there are various treatment approaches for asthma. On the basis of the GINA (Global Initiative for Asthma) and asthma management guidelines, ICS is considered the first-line treatment for asthma. Proper use of ICS, combined with peak flow monitoring, contributes to asthma control. ICS has a positive effect on managing asthma symptoms and can reduce airway inflammation and hyperresponsiveness [31]. For adult asthma patients, the recommended treatments variously depend on the severity of the condition. For mild asthma, regular low-dose ICS with Short-Acting Beta-Agonists (SABA) is the applicable choice. For moderate asthma, ICS-Formoterol is recommended based on substantial clinical evidence, while for severe asthma, ICS-Long-Acting Beta-Agonist (LABA) plus Long-Acting Muscarinic Antagonist (LAMA) or OCS may be necessary [32]. It’s important to note that LABA use as monotherapy should be avoided [33]. Exposure to OCS is crucial in managing asthma exacerbations and severe asthma. However, prolonged OCS usage raises the risk of OCS-related complications, including adverse effects on organs like bones, muscles, and skin [34].

In the past several years, biologic therapies have shown significant efficacy in asthma treatment. Omalizumab (anti-IgE), inhibits Th2 inflammation and reduces peripheral blood eosinophil counts in adolescents [35]. Other biologics such as Benralizumab (anti-IL-5Rα), Dupilumab (anti-IL-4Rα), Mepolizumab (anti-IL-5), and Reslizumab (anti-IL-5) have been used in asthma patients and have demonstrated positive therapeutic effects in some cases. However, due to the heterogeneity of asthma, their efficacy is not universally certain [36–38]. Asthma-panic disorder (PD) is relatively prevalent in the population, and both Music Relaxation Therapy (MRT) and Cognitive-Behavioral Psychophysiological Therapy (CBPT) have shown significant benefits in reducing blood pressure, respiratory rate, heart rate, and degree of anxiety while improving asthma symptom. Notably, CBPT stands out as more effective than MRT in enhancing compliance with ICS treatment [39]. Traditional Chinese Medicine (TCM), characterized by individualized and multi-meridian intervention, has shown promise in alleviating asthma symptoms and improving lung function. Studies have found that TCM interventions such as tonifying qi, tonifying the kidneys, and strengthening the spleen can improve symptoms, decrease the frequency of asthma acute exacerbation, and To alleviate airway resistance in children 2–5 years old with asthma [40]. However, clinical research on the satisfactory effectiveness of TCM is still limited, and its mechanism of action remains unclear. Further clinical applications on a global scale are needed.

## 4. MSCs

### 4.1. MSCs overview

MSCs, also known as multipotent stromal cells, can be gained from a range of tissues, including bone marrow, adipose tissue, umbilical cord blood, amniotic fluid, and others. They were first recognized by researchers as early as 1990 and have gradually gained recognition. The main sources of MSCs are Bone Marrow Mesenchymal Stem Cells (BM-MSCs), Adipose Tissue Mesenchymal Stem Cells (AD-MSCs), and Umbilical Cord Blood Mesenchymal Stem Cells (UCB-MSCs) [41]. while MSCs from different sources of origin may exhibit variations in phenotype and functional characteristics, they are defined by the minimal criteria established by the International Society for Cellular Therapy(ISCT): (1) MSCs express CD29, CD90, CD73, CD105, CD71, CD271, and do not express CD14, CD34, CD45, and human leukocyte antigen DR (HLA-DR); (2) they possess adhesive properties; (3) they have the ability to differentiate into mesodermal cell lineages such as adipocytes and osteoblasts [42]. MSCs possess not only tissue repair capabilities and immunosuppressive properties but can also release various bioactive factors, making them pivotal in allergic diseases [43]. They also exhibit low immunogenicity [44]. Due to the limited expression of MHC II molecules, Fas ligand (Fas L), and T cell co-stimulatory molecules on MSCs, they retain the ability to induce host immune suppression even in an allogenic environment [45].

MSCs have attracted attention for their ease of isolation and preservation, lack of significant ethical concerns, and reduced risk of tumorigenicity. Through releasing multifarious paracrine factors, such as EXOs, MSCs can promote the differentiation and proliferation of diverse cell types. MSCs have various effects, including anti-inflammation [46], anti-fibrosis [47], anti-apoptosis [48], antimicrobial [49], antioxidation [50], pro-angiogenesis [51], enhancement of alveolar fluid clearance [52], and improvement of endothelial and epithelial cell damage in the lungs [53]. Furthermore, MSCs possess “homing” abilities, allowing them to migrate to sites of injury [54]. These distinctive attributes position MSCs as a promising asset in the realm of regenerative medicine.

### 4.2. MSCs in the treatment of asthma

Originally, researchers believed that MSCs had the ability to differentiate into a variety of cell types, but now this is no longer regarded as the primary mechanism [55]. MSCs have the capacity to recognize their microenvironment and alter their phenotype and secretion profile to modulate the biological behavior of other cells [56–58]. MSCs engage with target cells through direct contact between cells. Concurrently, they can also release soluble mediators and EVs, thereby facilitating communication with other cells *via* paracrine/endocrine pathway. Additionally, MSCs can transfer mitochondria to damaged cells, enhancing their antioxidant function and thus repairing the injured cells. This is particularly meaningful since mitochondrial dysfunction is very common in chronic lung diseases such as chronic obstructive pulmonary disease (COPD), asthma, and idiopathic pulmonary fibrosis (IPF) [59,60]. As a prevalent chronic respiratory disease, asthma affects a substantial portion of the population. Globally, it is reported that about 5% to 12% of asthma patients are severely drug-resistant [61]. Therefore, there is still a need for new treatment approaches.

Several experimental studies have been published demonstrating the beneficial effects of MSCs on asthma. In one study, BM-MSCs were confirmed to exhibit immunoreaction in an asthmatic mouse through both intratracheal and systemic administration. The authors suggested that BM-MSCs treatment could be a potential approach to reduce eosinophil counts, inhibit the release of inflammatory cytokines, reduce excessive mucus production, and regulate mitochondrial dysfunction while increasing the levels of IFN-γ to prevent lung injury [62]. Shin et al. also evaluated the beneficial effects of UC-MSCs on a severe asthma mouse model, which showed improvement by suppressing Th2 cell inflammation [63]. Furthermore, *in vitro* experiments demonstrated that UC-MSCs could directly reduce the secretion of IL-5 and IL-13 in Th2 cells and peripheral blood mononuclear cells (PBMCs) from mice model of asthma. In an ovalbumin (OVA) -induced acute asthma rat model, intraperitoneal injection of placental mesenchymal stem cells (PMSCs) exhibited a beneficial impact on asthmatic rats by suppressing inflammation through the regulation of the Notch signaling pathway. Additionally, this study results indicated a noticeable increase in IFN-γ and a decrease in IL-4 and IgE levels following PMSC transplantation [64]. Recent research has pointed out that treatment with MSCs engineered to express the IL-35 or IL-10 gene can reduce cytokine levels (specifically IL-33, IL-4, IL-5, and IL-13) in bronchoalveolar lavage fluid (BALF) of asthmatic mice. This treatment also mitigates inflammatory reaction, the hyperplasia of goblet cells, and the secretion of mucus. The experiments demonstrated that treatment with MSC-IL-35 or MSC-IL-10 was more effective than using MSCs alone. This suggests that the immunomodulatory effects of MSCs synergize with the immunoregulatory effects of IL-35 and IL-10 to jointly regulate the progression of asthma inflammation [65,66].

## 5. MSC-EXOs

### 5.1. MSC-EXOs overview

EXOs can originate from various types of cells, including immune cells, stem cells, epithelial cells, and more. These extracellular vesicles are released through the fusion of multivesicular bodies with the cell membrane, typically having a diameter ranging from 30 to 150 nm. EXOs serve as mediators for intercellular communication and can transport nucleic acids (such as DNA, mRNA, and miRNA), proteins, enzymes, and metabolites, thereby altering the biological functions of target cells [67]. They play an important part in various physiological and pathological responses, including cell proliferation, inflammation, immune regulation, angiogenesis, and changes in endothelial permeability. Under various physiological and pathological conditions, EXOs can be discharged into the extracellular space and various body fluids, including blood, cerebrospinal fluid, semen, bile, amniotic fluid, ascites, and bronchoalveolar lavage fluid [68]. They have been widely studied and reported to be closely associated with various inflammatory and respiratory diseases. As a result, EXOs have garnered significant attention as potential new biomarkers and therapeutic targets.

Moreover, EXOs are an essential component of the paracrine secretion pathway of MSCs and can exert therapeutic effects similar to their parent cells. They carry important cell factors that can modulate inflammation, immune responses and cellular biological functions [69]. While BM-MSCs are the most common cell source for the preparation of EXOs, recent studies have proved that induced pluripotent stem cell (iPSC)-derived MSCs exhibit phenotypic and functional similarities to adult MSCs [70]. They demonstrate longer survival time, higher proliferation potential, stronger immunomodulatory properties, lower heterogeneity, and do not induce tumor formation, making them a perfect cell source for the large-scale production of EXOs [71].

Compared to cell-based therapies using MSCs, MSC-EXOs offer several advantages, including low immunogenicity, ease of storage, and high biocompatibility. They do not have the risk of tumor formation since they cannot replicate autonomously. They also have a longer shelf life through sterile filtration, and long-term repeated administration of Exos does not induce toxicity [72]. To date, two clinical studies have reported the use of MSC-EXOs for the treatment of graft-versus-host disease (GVHD) and chronic kidney disease (CKD) patients without severe side effects [73,74] Therefore, MSC-EXOs are considered highly promising cell-free therapeutic agents, potentially harnessing the functions of entire cells while overcoming many common issues associated with live cell therapy. This offers new opportunities for the diagnosis and treatment of asthma and other respiratory diseases.

### 5.2. The mechanisms of action of MSC-EXOs therapy in asthma (Figure 2)

#### 5.2.1. Inhibit airway inflammation and immune responses

The immune response of T lymphocytes plays an important role in the development of asthma, and MSC-EXOs can influence the physiological activities of T Lymphocytes. Firstly, MSC-EXOs increase the proportion and functionality of regulatory T cells (Tregs). Tregs are effective immune regulators characterized by the expression of the transcription factor Foxp3, and they suppress allergic airway inflammation by inhibiting allergic responses [75–77]. In a recent study, we found that MSC-EXOs target c-Jun *via* miR-1470, inducing the expression of P27KIP1, which enhances the proportion of Tregs in the peripheral blood of asthma patients. This, in turn, suppresses the immune response and improves airway remodeling in asthma [78]. In another study evaluating the immunomodulatory effects of MSC-EXOs on PBMCs of asthma patients, the data showed that MSC-EXOs increased the secretion of IL-10 and TGF-β1 by PBMCs, thereby enhancing the proliferation and immunosuppressive ability of Tregs and exerting an immunomodulatory effect in asthmatic mice [79]. Moreover, a recent study found that intranasal injection of AD-MSC-EVs significantly reduced allergic airway inflammation in asthmatic mice by inducing an increase in Tregs in the blood, improving AHR [80]. However, they couldn’t distinguish whether these effects were related to exosomes, microvesicles, or their binding.

Furthermore, MSC-EXOs can modulate the Th1/Th2 balance. The significant immunological mechanism in the development of allergic asthma is the dominance of Th2 cells resulting from an imbalance in the Th1/Th2 ratio [81]. Cytokines secreted by Th1 cells (IL-2 and IFN-γ) can inhibit the differentiation of Th2 cells and the recruitment and activation of macrophages and neutrophils. In contrast, cytokines secreted by Th2 cells (such as IL-4, IL-13, and IL-5) can increase the proliferation and recruitment of inflammatory cells [82]. Upregulating the Th1 cell phenotype is considered protective against allergic inflammatory reactions. Zhou et al. found that BM-MSC-EXOs could inhibit the differentiation of Th2 cells by regulating the miR-146a-5p/SERPINB5 signaling pathway [83]. This prompted us to think about asthma, which is also an allergic reaction. Dehnavi et al. found that sublingual immunotherapy with allergen-loaded MSC-EXOs reduced IgE levels in the serum and nasopharyngeal lavage fluid (NALF) of OVA-induced asthmatic mice, as well as IL-4, inflammatory cells, and eosinophil numbers, while increasing IFN-γ levels [84]. This shift from Th2 to Th1 suggests that exosome-based immunotherapy may help regulate allergic responses. Similarly, Asadirad et al. also found that administering allergen-rich MSC-EXOs sublingually to mice as a preventive measure modulated the immune response and suppressed lung inflammation in asthma [85]. These findings indicate that MSC-EXOs play an important part in modulating T lymphocytes responses in asthma and have the potential to be used as immunomodulatory therapies.

##### 5.2.1.1. Regulation of macrophage phenotype

Macrophages, comprising approximately 70% of all immune cells in lung tissue, hold a pivotal role in regulating airway inflammation and airway remodeling [86,87]. These immune cells can be classified into two primary activation types, namely classical activation (M1) and alternative activation (M2), based on specific cytokine expression and surface markers [88]. In cases of severe steroid-resistant asthma (SSRA) exacerbations, M1 macrophages have been observed to release substantial quantities of inflammatory mediators, including TNF-α, IL-6, IL-1β, and so on [89]. These inflammatory factors are linked to AHR and airway remodeling [90]. Conversely, M2 macrophages, identifiable by markers such as arginase-1 (Arg-1), IL-10, and CD206, create an anti-inflammatory environment that supports tissue repair [91]. Consequently, targeting the transition between M1 and M2 phenotypes could offer a novel therapeutic approach for SSRA treatment.

Li et al. confirmed that exosomes derived from M2 macrophages carrying miR-370 alleviate asthma progression by downregulating the MAPK/STAT1 signaling pathway [92]. Dong et al. conducted a study showing that intratracheal instillation of UC-MSC-EXOs in SSRA mice reversed inflammation, AHR, and histopathological changes [93]. *In vitro* results from this study also reported that MSC-EXO therapy promoted M2 polarization and suppressed M1 polarization in LPS-stimulated RAW 264.7 cells [93]. This mechanism might involve the regulation of the NF-κB and PI3K/AKT signaling pathways by targeting the expression of tumor necrosis factor receptor-associated factor 1 (TRAF1) [93]. Another animal study indicated that AD-MSC-EXOs modified by mmu_circ_0001359 seemed to enhance FoxO1 signaling pathway-mediated M2 macrophage activation through sponge-absorbing miR-183-5p, thereby reducing airway remodeling [94].

New research on the mechanisms underlying severe asthma suggests that lung macrophages play a significant role in the development of allergic airway inflammation. Fang et al. found that MSC-EXOs could be taken up by pulmonary alveolar macrophages (MoAMs) derived from monocytes *in vivo*, leading to the inhibition of MoAM differentiation and M2 macrophage polarization. This resulted in an improvement in predominantly TH2-mediated allergic airway inflammation and immune regulation [95]. Recently, there has been a growing focus on interstitial macrophages (IMs), whose role is crucial in maintaining lung homeostasis and preventing immune-mediated allergic airway inflammation. Ren et al. found that intranasal delivery of MSC-EXOs increased the proportion of lung IMs and enhanced constitutive IL-10 expression in IMs, offering significant protection in asthmatic mice [96]. Other studies have also found that MSC-EXOs (miR-21, miR-98) could increase IL-10 secretion in macrophages and induce JAK1 phosphorylation and STAT3 activation, while exosomes (miR-146a) could reduce intracellular iNOS production by inhibiting IRF5, thereby promoting the M2 macrophage phenotype [97]. These research findings collectively suggest that inducing M2 macrophages may be a viable strategy for treating asthma.

##### 5.2.1.2. Inhibiting the activation of ILC2s

Recent research has highlighted the significant role of Type 2 innate lymphoid cells (ILC2s) in allergic diseases. ILC2s are crucial innate immune cells, and their increased numbers are significantly associated with the release of cytokines such as IL-5, IL-9, and IL-13, as well as epithelial cell-derived cytokines like IL-25, IL-33, and thymic stromal lymphopoietin (TSLP). They play a pivotal role in initiating and sustaining type 2 allergic airway inflammation [98–100]. Additionally, in severe asthma cases, the abundance of ILC2s is related to persistent eosinophilic inflammation. Previous studies have found a significant increase in peripheral ILC2s in asthma and allergic rhinitis patients [101,102]. Fang et al. have also discovered, in mouse models and *in vitro* experiments, that small extracellular vesicles (small EVs) derived from human induced pluripotent stem cell-derived mesenchymal stem cells (iPSC-MSCs) could inhibit the activation of ILC2s, reduce inflammation, decrease lung mucus production, and lower AHR [103]. This effect was associated with the transfer of miR146a-5p from MSC-EVs to ILC2s. Therefore, inhibiting the activation of ILC2s appears to be a novel target in asthma treatment.

#### 5.2.2. *Delivering mitochondria enhances antioxidant capabilitie*s

Recent studies have suggested a connection between mitochondrial dysfunction in epithelial cells and allergic airway inflammation. Targeting mitochondrial dysfunction has emerged as a potential approach in the treatment of allergic airway inflammation. Notably, it has been reported that the mitochondrial-targeted antioxidant mitoTEMPO mitigated allergic asthma induced by OVA by reducing mitochondrial ROS levels in epithelial cells [104]. Thus, addressing mitochondrial dysfunction could be a viable avenue for managing allergic airway inflammation.

Moreover, recent research has indicated that MSC-EXOs, in addition to transporting bioactive substances, also play a role in mitochondrial transfer [105–107]. BM-MSCs transfer mitochondria-containing EXOs to peripheral T cells, thereby modulating T cell immune responses in asthma [107]. EXOs containing mitochondria from MSCs can also promote the polarization and oxidative phosphorylation of M2 macrophages, enhancing their bioenergetics [106]. The miRNAs carried by MSC-EXOs lead to increased expression of enzymes such as catalase, superoxide dismutase, and glutathione peroxidase, resulting in reduced reactive oxygen species production and enhanced antioxidant capabilities. Furthermore, MSC-EXOs have been shown to improve mitochondrial function, thereby reversing oxidative stress in epithelial cells [108,109]. These recent findings highlight that MSC-EXOs promote oxidative functionality in epithelial cells, regulate the bioenergetics of target cells, and hold promise in clinically restraining the progression of asthma.

#### 5.2.3. Ways to improve airway remodeling

Airway remodeling is a major characteristic of asthma, characterized by changes such as collagen deposition, increased mucous gland hyperplasia, increased smooth muscle quantity, and goblet cell hyperplasia [110,111]. MSC-EXOs mitigate airway remodeling by limiting goblet cell hyperplasia, subepithelial smooth muscle hyperplasia, and collagen deposition.

Firstly, EXOs can act on the TGF-β/Smad signaling pathway: TGF-β is a pro-fibrotic cytokine, and the activation of the TGF-β/Smad signaling pathway plays a central role in the development of airway remodeling, making it a potential therapeutic target for asthma [112]. TGF-β1 can downregulate the expression of E-cadherin in bronchial epithelial cells, induce fibroblasts to transform into myofibroblasts, promote the expression of α-smooth muscle actin (α-SMA), and induce epithelial-mesenchymal transition (EMT) in bronchial epithelial cells [113,114]. Dong et al. discovered that UC-MSC-EXOs under hypoxic conditions exhibited greater effectiveness in restraining the release of pro-inflammatory cytokines like IL-4 and IL-13 in an OVA-induced asthma mouse model [115]. This led to a reduction in eosinophil count, decreased levels of α-SMA and collagen-1, inhibition of the TGF-β1-p-smad2/3 signaling pathway expression, and a decrease in airway remodeling [115]. Additionally, UC-MSC-EXOs displayed enhanced anti-inflammatory and anti-fibrotic properties.

Another significant impact of EXOs is their modulation of the Wnt/β-catenin pathway. This pathway, activated by the binding of the ligand protein Wnt to its membrane receptor, plays a crucial part in regulating the development of chronic asthma-related airway remodeling [116]. In recent studies, it has been demonstrated that BM-MSC-EXOs miR-188 can effectively diminish the proliferation of bronchial smooth muscle cells and mitigate lung injury in the OVA-induced asthma mouse model by attenuating the JARID2/Wnt/β-catenin axis. This intervention leads to the inhibition of asthma’s pathological progression [117]. Additionally, Song et al. have reported that MSC-EXOs can reduce airway remodeling and EMT by suppressing the Wnt/β-catenin signaling pathway [118]. Li et al. have uncovered the pivotal role of BM-MSC-EXOs miR-223-3p in airway remodeling and asthma protection by modulating the NLRP3-induced ASC/Caspase-1/GSDMD signaling pathway [119]. In a similar vein, research has indicated that AD-MSC-EXOs enriched with miR-301a-3p effectively alleviate platelet-derived growth factor (PDGF)-BB-induced remodeling and inflammation in ASMCs by targeting STAT3 [120]. Additionally, Liu et al. have reported that BM-MSC-EXO carrying miR-221-3p inhibit the expression of fibroblast growth factor 2 (FGF2) and the ERK1/2 signaling pathway, resulting in reduced proliferation and migration of ASMCs, decreased extracellular matrix (ECM) deposition, and alleviation of airway remodeling in OVA-induced asthma mice [121]. These findings underscore the diversity of signaling pathways through which MSCs enhance asthma remodeling, offering valuable insights for the development of therapeutic strategies to mitigate asthma recurrence.

MSC-EXOs, as a cell-free modality of regenerative medicine, not only hold substantial promise in ameliorating airway remodeling in asthma but also demonstrate considerable potential in facilitating the repair and remodeling of various tissues and organs. For instance, in the context of myocardial ischemia/reperfusion (I/R) injury, MSC-EXOs have been shown to attenuate cardiomyocyte apoptosis, fibroblast proliferation, and inflammatory responses, thereby improving cardiac function, reducing myocardial remodeling, and mitigating the increased risks of arrhythmias or hepatic, renal, or cardiac toxicity, thereby offering novel therapeutic targets and strategies for ischemic heart disease [122]. Moreover, MSC-EXOs play an essential role in the therapeutic management of gastrointestinal disorders. For example, AD-MSC-EXOs contribute significantly to the remodeling of colonic mucosal cell structures by elevating the levels of anti-inflammatory factors, suppressing pro-inflammatory factors, and promoting the regeneration and proliferation of intestinal stem cells, epithelial cells, and goblet cells within the colonic crypts, thereby maintaining mucosal integrity [123]. Yi et al. [124] demonstrated that both MSC-EXOs and MSCs promote tissue repair in rats with liver injury through mechanisms such as the elevation of M2 macrophages, alleviation of inflammation, and modulation of various intestinal microbiota metabolic pathways, with MSC-EXOs exhibiting superior effects compared to MSCs. Moreover, numerous studies have substantiated the potential therapeutic effects of MSC-EXOs in promoting cartilage repair, fracture healing, and skin tissue regeneration. Zhang et al. [125] developed a bioactive 3D porous polylactic acid-exosome scaffold (PLA-EXO), and the study revealed that this scaffold significantly attenuated the production of pro-inflammatory factors, enhanced matrix mineralization and calcium deposition in BM-MSCs, and facilitated bone regeneration through the regulation of cytokine and chemokine secretion. In the context of chronic wound repair, MSC-EXOs cultured under three-dimensional (3D) conditions promoted fibroblast proliferation and keratinocyte migration, thereby reducing the area of granulation tissue. Compared to MSC-EXOs cultured under static two-dimensional (2D) conditions, those cultured in 3D conditions exhibited enhanced capacity to deliver repair-related proteins and modulate immune cell functions, significantly promoting skin wound healing both *in vitro* and *in vivo* [126]. Excessive fibroblast activation underlies the process of wound repair and scar formation. Owing to their acellular nature, as well as their immunomodulatory and regenerative properties, MSC-EXOs have attracted significant attention in the treatment of pathological scars. Specifically, MSC-EXOs expedite the healing process and improve healing quality by enhancing glucose and lipid utilization in wound tissues, while regulating fibroblast amino acid metabolism to inhibit their proliferation, differentiation, and collagen synthesis. Moreover, MSC-EXOs influence amino acid recycling and utilization during the healing process through the modulation of autophagy, thereby mitigating abnormal scar formation [127].

## 6. Summary and limitations

Addressing airway damage in asthma has been a long-standing and formidable challenge within the realm of pulmonary regenerative medicine. Traditional treatment methods and MSC cell therapies both have their limitations. Existing research results suggest that MSC-EXOs have roles in suppressing inflammatory responses, participating in immune regulation, improving airway epithelial remodeling, and slowing the progression of asthma. They may offer a safer and more cost-effective approach compared to MSC cell therapy. Nevertheless, our comprehension of the underlying molecular mechanisms remains constrained, and the field lacks comprehensive large-scale, randomized, placebo-controlled clinical trials to gauge the true impact of MSC-EXOs on asthma. As a cell-free alternative therapy, research on MSC-EXOs is still in its early stages, and several questions need to be addressed, including:

- Which type of MSC is the optimal source for preparing EXOs?
- What is the most effective route of administration, systemic (intravenous, intraperitoneal) or local (intratracheal, intrapleural, intranasal)?
- Regarding administration frequency, is a single administration superior, or is multiple administrations more effective?
- Which components play a key role in the biological effects of MSC-EXOs?
- What is the gold standard animal model for extracting MSC-EXOs?

Therefore, extensive clinical research is still required in the future to delve deeper into the effectiveness, mechanisms, and safety of MSC-EXOs in asthma treatment in humans.

## Data Availability

Data sharing is not applicable to this article as no new data were created or analysed in this study.
